# Characteristics and direct causes of death in severely ill patients admitted to a neurology department

**DOI:** 10.3389/fneur.2024.1386403

**Published:** 2024-12-13

**Authors:** Da Heui Lee, Seok Young Jeong, Byoung-Soo Shin, Hyun Goo Kang

**Affiliations:** ^1^Jeonbuk National University Medical School, Jeonju, Republic of Korea; ^2^Department of Neurology, Jeonbuk National University Medical School and Hospital, Jeonju, Republic of Korea; ^3^Research Institute of Clinical Medicine of Jeonbuk National University, Biomedical Research Institute of Jeonbuk National University Hospital, Jeonju, Republic of Korea

**Keywords:** death, neurology, stroke, cause of death, hospitalization

## Abstract

**Introduction:**

The study aimed to analyze the characteristics of patients admitted to the neurology department of a tertiary hospital who subsequently died, focusing on those with high disease severity.

**Materials and methods:**

We conducted a retrospective cohort study of patients who died among those admitted to the neurology department of a regional tertiary hospital from 2013 to 2021. Clinical, radiological, and laboratory results of the included patients were collected, and their primary diagnoses, duration from time of admission to death, and direct causes of death were analyzed. Furthermore, the patients were categorized into subgroups based on sex (male and female), primary diagnosis (ischemic and non-ischemic stroke), and cancer diagnosis for comparative analysis.

**Results:**

Of 187 deaths, the primary diagnoses were ischemic stroke (131 cases), seizures (19 cases), encephalitis and encephalopathy (18 cases), and other conditions (19 cases). The direct causes of death included ischemic stroke in 68 patients, sepsis in 33, cerebral hemorrhage in 19, pneumonia in 15, acute kidney injury in nine, status epilepticus in seven, and other causes in 36. Pneumonia, cerebral hemorrhage, acute kidney injury, and status epilepticus were the more prevalent direct causes of death in men, whereas ischemic stroke and sepsis were more prevalent in women. Additionally, sepsis, pneumonia, acute kidney injury, and status epilepticus, as direct causes of death, were significantly higher among patients with a primary diagnosis of non-ischemic stroke than among those with a primary diagnosis of ischemic stroke. Furthermore, there were differences in some pre-existing diseases and laboratory findings when comparing between the cancer group and the non-cancer group.

**Discussion:**

Ischemic stroke was the primary diagnosis and direct cause of death in a high proportion of patients. Other noteworthy direct causes of death were cerebral hemorrhage and infections such as sepsis and pneumonia. Based on these findings, the characteristics and prognoses of patients admitted to neurology departments can be predicted and used for management.

## 1 Introduction

Several neurological diseases, such as stroke, Alzheimer’s disease and other dementias, Parkinson’s disease, epilepsy, and headache (migraine, tension-type headache, and medication-overuse headache), are significant health problems suffered by individuals worldwide (1). These neurological diseases have become increasingly important, as they cause a significant proportion of deaths in the modern world.

In September 2019, the World Health Organization (WHO)’s list of the top ten causes of death showed that stroke was the second leading cause of death; Alzheimer’s disease and other dementias ranked seventh. Moreover, since 2000, stroke has been considered an important neurological disease with a high incidence rate (2). Although Alzheimer’s disease and other dementias showed a low incidence percentage, it was considerably higher than that in previous studies, thus Evidencing the importance of neurological diseases in modern society (2). Previous studies mainly focused on a specific neurological disease. For example, Yuan et al. (3) and Diaz et al. (4) analyzed the characteristics of patients with stroke, such as the immediate causes of death.

Therefore, the present study focused on all deaths among hospitalized patients and aimed to holistically study the cases of patients with neurological diseases rather than focusing on the characteristics and specific diseases of neurological disease-related deaths in South Korea, an East Asian country. Specifically, this study aimed to examine the characteristics and deaths of patients with severe diseases who visited a regional tertiary hospital rather than those of patients with low-severity diseases at primary and secondary hospitals. Additionally, this study aimed to examine the progression of neurological conditions leading to death in patients with high disease severity by investigating the direct causes of death, irrespective of the primary diagnosis that necessitated hospitalization. As a result, we expected that there would be significant differences between direct causes of death if the patients’ main diagnosis was different.

## 2 Methods

### 2.1 Patient selection

We conducted a retrospective cohort study of the cases of 187 patients who died in the neurology department of a regional tertiary hospital over nine years (2013–2021). The primary diagnosis, direct cause of death, indirect cause of death, main complaints at admission, medical history, diagnoses during hospitalization, stroke risk factors, and laboratory findings were collected from on electromagnetic resonance (EMR) records. Moreover, the above items were compared between men and women, patients with ischemic stroke and those with non-ischemic stroke, and patients with and without cancer. The methodologies used in this study complied with the STROBE guidelines (5).

### 2.2 Definitions

In the principal diagnostic classification, ischemic stroke included confirmed acute ischemic stroke on brain magnetic resonance imaging (MRI). Seizures included epilepsy, status epilepticus, and convulsive seizures, which were not otherwise specified. Epilepsy was defined as the occurrence of two or more unprovoked seizures at least 24 h apart. Status epilepticus was defined as (1) persistent clinical or electroencephalogram seizure activity or (2) recurrent seizure activity lasting >5 min, with no recovery period between seizures. Meningoencephalitis was defined as a central nervous system infection involving the brain, and was classified into viral, bacterial, tuberculous, and Listeria meningoencephalitis. Encephalopathy included Wernicke encephalopathy and metabolic encephalopathy.

As a direct cause of death, sepsis was defined as a state of organ dysfunction in which the total sequential organ failure assessment (SOFA) score acutely changed by two or more points due to infection (6). Pneumonia was identified as a direct cause of death in a case with clinical (crackles and new purulent sputum) or imaging (new or progressive pulmonary infiltration, pulmonary sclerosis, cavitation, and pleural effusion) evidence of pneumonia along with fever or leukocytosis. Patients with no evidence of pneumonia were excluded after rechecking their EMR records. Cerebral hemorrhage was defined as a hemorrhage confirmed using computed tomography (CT) or MRI. Acute kidney injury was defined as an increase in creatinine (Cr) level of 0.3 mg/dL during hospitalization or an increase of at least 50% from baseline.

Furthermore, we measured the time from hospitalization to death and examined other stroke risk factors (hypertension, diabetes mellitus, atrial fibrillation, dyslipidemia, alcohol consumption, smoking history, and stroke history) at the time of admission. Hypertension was defined as a blood pressure of 140/90 mmHg or higher at rest during hospitalization (7). Diabetes mellitus was defined as a blood glucose level of 200 mg/dL or higher after 2 h on an oral glucose tolerance test, a fasting blood glucose level of 126 mg/dL or higher, a hemoglobin A1c (HbA1c) level of 6.5% or higher (8), or use of diabetes medication. Atrial fibrillation was diagnosed when atrial fibrillation or atrial flutter was confirmed on an electrocardiogram (ECG). Dyslipidemia was defined as low-density lipoprotein (LDL) level of 160 mg/dL or higher, total cholesterol level of 240 mg/dL or higher, and triglycerides (TG) level of 200 mg/dL or higher. Patients were considered to have a history of drinking or smoking if they had consumed alcohol or smoked within the last 5 years. Previous diseases were categorized into disease groups based on similarities using disease names and codes, according to the International Classification of Diseases 11th Revision (ICD-11). In addition, we investigated whether patients had a history of tracheal intubation or hospitalization in the intensive care unit (ICU).

This study investigated laboratory findings showing general blood count, electrolyte levels, liver function tests, and inflammation (i.e., white blood cell, hemoglobin, platelet, erythrocyte sedimentation rate, sodium, potassium, albumin, alanine aminotransferase, aspartate aminotransferase, alkaline phosphatase, and C-reactive protein), those related to blood coagulation that could affect ischemic stroke (i.e., prothrombin time, activated partial thromboplastin time, D-dimer, fibrinogen, and fibrinogen degradation products), and those evaluating the patient’s underlying condition at the time of admission (i.e., blood urea nitrogen, creatinine, hemoglobin A1c, triglyceride, high-density lipoprotein, LDL, total cholesterol, calcium, thyroid-stimulating hormone, and free T4) based on blood tests at the time of admission.

Fazekas scale was used to describe hyperintense signal abnormalities in the deep white matter. White matter hyperintensity (Fazekas scale) was measured as follows: 0 = absent, 1 = punctate foci, 2 = beginning confluence of foci, and 3 = large confluent areas (9). The microbleeds refer to hypointensive small, round, or ovoid spots which are <10 mm in diameter, found on susceptibility-weighted imaging (SWI). Microbleeds were classified into absent, mild (total count 1 to 2), moderate (3 to 10), and severe (>10) groups (10).

### 2.3 Statistical analysis

We compared the demographic and laboratory findings of male and female patients who died in the neurology department. Furthermore, we conducted comparative analyses of cases in which the cause of death was ischemic or non-ischemic stroke. Similarly, we categorized patients into those with and those without cancer for comparative analysis. Pearson’s chi-square or Fisher’s exact tests were used for categorical variables and the t-test was used for continuous variables. Statistical significance was set at *p* < 0.05 (two-tailed). Results of multivariate logistic regression analysis were presented as *p*-values, adjusted odds ratios (aOR), and 95% confidence intervals (CI). To identify factors associated with death from ischemic stroke, sepsis, and pneumonia, we conducted univariate logistic regression analysis, selecting independent variables with *p*-values ≤0.1, followed by multivariate logistic regression analysis. All statistical analyses were performed using the Statistical Package for the Social Sciences (SPSS) 21.0 (IBM Corp., Armonk, NY, USA).

## 3 Results

**Figure 1:**
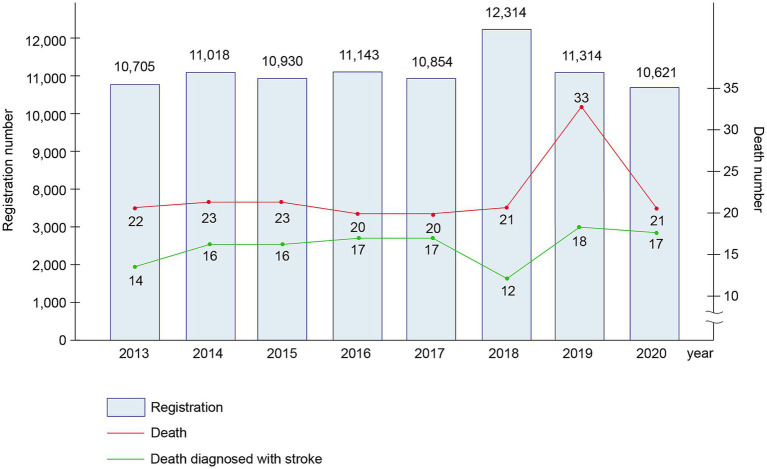
Number of patient registrations and deaths from 2013 to 2020.

From 2013 to 2020 (eight years), 88,899 patients were hospitalized at our neurology department; of these, 187 patients died. Figure 1 shows the annual number of deaths. Of the 187 deceased patients, 96 (51.3%) were men and 91 (48.7%) were women, with a mean age of 74.35 ± 12.63 years (Supplementary Table 1). Hypertension was the most common risk factor of stroke (65.8%), followed by diabetes mellitus (32.6%), atrial fibrillation (27.3%), smoking (26.7%), alcohol consumption (24.6%), and dyslipidemia (8.0%). Among the deceased patients, 146 (78.1%) underwent tracheal intubation during hospitalization and 152 (81.3%) received care in the ICU (Supplementary Table 1).

**Figure 2:**
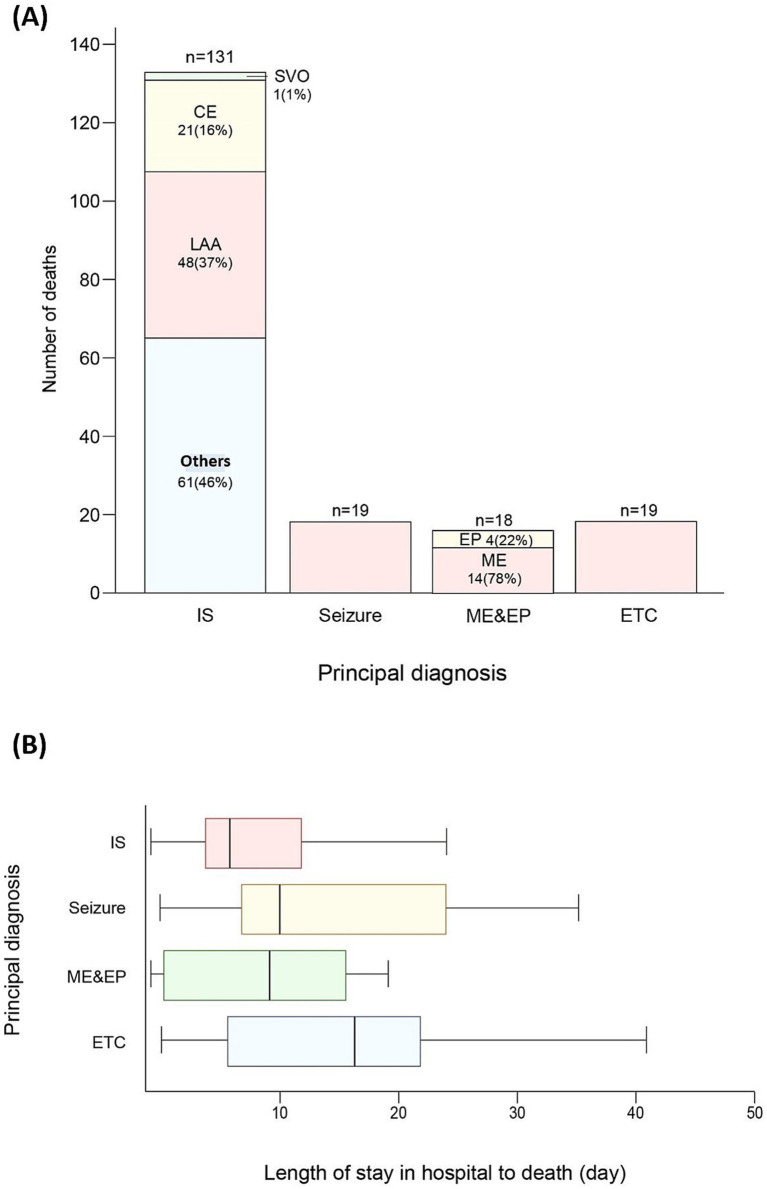
**(A)** Number of deaths resulting from principal diagnoses. **(B)** Comparison of length of stay in hospital to time of death from principal diagnoses. Lower and upper hinges of boxes represent the first and third quartiles for length of stay in hospital to time of death, respectively. Central lines in the boxes correspond to median values. The upper and lower whiskers extend to the largest and smallest values no further than 1.5 × IQR from each hinge, respectively. Outliers are excluded. IS, ischemic stroke; LAA, large artery atherosclerosis; CE, cardioembolism; SVO, small-artery occlusion; ME, meningoencephalopathy; EP, encephalopathy ETC as a principal diagnosis includes malignant neoplasm (4), brain abscess (3), tetanus (3), acute transverse myelitis (2), IgG4-related disease (1), injury of cervical spinal cord (1), halogenated insecticides (1), anoxic brain damage (1), Guillain-Barre syndrome (1), Creutzfeldt-Jakob disease (1), myocardial infarction (1). Others in ischemic stroke contain stroke of other determined etiology and stroke of undetermined etiology.

The primary diagnoses of the patients who died included ischemic stroke (131 patients, 70.1%), seizures (19 patients, 10.2%), encephalitis or encephalopathy (18 patients, 9.5%), and other diagnoses (19 patients, 10.2%). According to the TOAST classification, ischemic stroke cases included large artery atherosclerosis (48 patients, 37%), small-artery occlusion (one patient, 1%), cardioembolism (21 patients, 16%), and others (61 patients, 46%). Moreover, regarding encephalitis and encephalopathy, 14 patients had encephalitis (78%) and four had encephalopathy (22%) (Supplementary Table 1 and Figure 2A). The direct causes of death were ischemic stroke (68 patients, 36.4%), sepsis (33 patients, 17.6%), pneumonia (15 patients, 8.0%), cerebral hemorrhage (19 patients, 10.2%), acute kidney injury (nine patients, 4.8%), status epilepticus (seven patients, 3.7%), and other causes (36 patients, 19.3%). On average, the patients had a time of 13.13 ± 22.57 days between hospitalization and death. The mean time from hospitalization to death was 11.31 ± 22.87 days for patients with ischemic stroke, 16.37 ± 16.02 days for those with a seizure, and 12.83 ± 18.47 days for those with encephalitis and encephalopathy (Supplementary Table 1 and Figure 2B).

The patients who died after hospitalization in the neurology department were categorized according to sex and compared (Table 1). The mean age was 72.17 ± 12.83 in men and 76.65 ± 12.06 in women (*p* = 0.015). For the direct causes of death, Male patients had significantly higher frequencies of pneumonia (10.4 vs. 5.5%), cerebral hemorrhage (16.7 vs. 3.3%), acute kidney injury (7.3 vs. 2. 2%), and status epilepticus (5.2 vs. 2.2%) than did the female patients (*p* = 0.001), whereas the female patients had significantly higher occurrences of ischemic stroke (26.0 vs. 47.3%) and sepsis (14.6 vs. 20.9%) than did the male patients (*p* = 0.001). Regarding stroke risk factors, hypertension (58.5 vs. 74.7%, *p* = 0.020) and atrial fibrillation (20.4 vs. 36.4%, *p* = 0.017) were significantly more common in women than in men, whereas smoking (45.8 vs. 6.6%, *p* < 0.001) and alcohol consumption (44.8 vs. 3.3%, *p* < 0.001) were significantly more common in men than in women. The other factors were not significantly different between sexes.

**Table 1:** Comparison of patients according to sex.

	Male (*n* = 96)	Female (*n* = 91)	*p* value
Age, mean ± standard deviation (years)	72.17 ± 12.83	76.65 ± 12.06	0.015
With Ischemic stroke	64 (66.7)	67 (73.6)	0.299
Principal diagnosis classification			
Ischemic stroke	64 (66.7)	67 (73.6)	0.201
Seizure	9 (9.4)	10 (11.0)	
Encephalitis and encephalopathy	8 (8.3)	10 (11.0)	
ETC*	15 (15.6)	4 (4.4)	
Direct cause of death			
Ischemic stroke	25 (26.0)	43 (47.3)	0.001
Sepsis	14 (14.6)	19 (20.9)	
Pneumonia	10 (10.4)	5 (5.5)	
Cerebral hemorrhage	16 (16.7)	3 (3.3)	
Acute kidney injury	7 (7.3)	2 (2.2)	
Status epilepticus	5 (5.2)	2 (2.2)	
ETC	19 (19.8)	17 (18.6)	
Length of hospital day	13.37 ± 17.34	12.89 ± 27.03	0.887
Hypertension	55 (58.5)	68 (74.7)	0.020
Diabetes mellitus	30 (31.9)	31 (34.4)	0.716
Atrial fibrillation	19 (20.4)	32 (36.4)	0.017
Smoking	44 (45.8)	6 (6.6)	<0.001
Dyslipidemia	5 (5.4)	10 (11.4)	0.150
Alcohol	43 (44.8)	3 (3.3)	<0.001
Body mass index (kg/m^2^)	22.66 ± 2.86	22.92 ± 4.10	0.859
Previous stroke history	22 (24.2)	21 (23.3)	0.894
Pre-existing disease			
Cancer	18 (18.8)	12 (13.2)	0.300
Circulatory	20 (20.8)	15 (16.5)	0.446
Nervous	11 (11.5)	11 (12.1)	0.894
Dementia	5 (5.2)	13 (14.3)	0.035
Genitourinary	12 (12.5)	19 (20.9)	0.124
Musculoskeletal, Connective tissue	9 (9.4)	16 (17.6)	0.099
Respiratory	8 (8.3)	12 (13.2)	0.283
Digestive	7 (7.3)	9 (9.9)	0.525
Endocrine	1 (1.0)	6 (6.6)	0.046
Others	24 (25.0)	16 (17.6)	0.216
Tracheal intubation	75 (80.6)	71 (78.0)	0.660
Intensive care unit care	78 (88.6)	74 (83.1)	0.294
White matter hyperintensity (Fazekas scale)			
0	2 (2.9)	0	0.208
1	22 (32.4)	17 (30.9)	
2	14 (20.6)	19 (34.5)	
3	30 (44.1)	19 (34.5)	
Microbleeds			
None	1 (1.5)	3 (4.4)	0.214
Mild	7 (10.3)	7 (10.3)	
Moderate	27 (39.7)	36 (52.9)	
Severe	33 (48.5)	22 (32.4)	
Laboratory finding			
WBC ($10^{3}$/μL)	11.04 ± 4.58	11.59 ± 5.77	0.475
Hemoglobin (g/dL)	12.16 ± 2.87	11.92 ± 2.04	0.504
Platelet ($10^{3}$/μL)	213.15 ± 98.61	217.97 ± 96.54	0.737
ESR (mm/h)	31.48 ± 26.08	31.78 ± 27.83	0.939
Sodium (mEq/L)	136.57 ± 4.85	137.85 ± 5.35	0.092
Potassium (mEq/L)	4.60 ± 2.95	4.10 ± 0.75	0.116
C-reactive protein (mg/dL)	39.61 ± 57.62	48.48 ± 77.62	0.380
Albumin (g/dL)	3.70 ± 0.80	3.76 ± 0.67	0.583
AST (U/L)	51.73 ± 89.44	42.93 ± 34.88	0.382
ALT (U/L)	38.64 ± 53.16	26.90 ± 22.15	0.053
ALP (U/L)	110.86 ± 84.45	111.20 ± 120.80	0.987
BUN (mg/dL)	25.11 ± 13.87	24.93 ± 17.93	0.942
Creatinine (mg/dL)	2.39 ± 11.87	1.09 ± 0.96	0.299
HbA1c (%)	6.12 ± 1.19	6.45 ± 1.32	0.118
Triglycerides (mg/dL)	119.06 ± 90.21	145.27111.12	0.203
HDL (mg/dL)	36.85 ± 15.40	38.76 ± 14.31	0.531
LDL (mg/dL)	88.58 ± 41.28	93.93 ± 41.42	0.524
Total cholesterol (mg/dL)	141.27 ± 48.12	154.85 ± 48.64	0.166
Calcium (mg/dL)	8.73 ± 0.73	8.22 ± 1.67	0.107
TSH (μIU/mL)	2.05 ± 2.92	3.76 ± 9.27	0.156
Free T4 (mg/dL)	17.08 ± 8.39	16.04 ± 4.27	0.397
Fibrinogen (mg/dL)	325.87 ± 90.21	346.98 ± 125.20	0.314
FDP (ug/mL)	31.85 ± 100.70	17.97 ± 28.27	0.316
D-dimer (mg/L FEU)	3.91 ± 6.00	3.81 ± 5.17	0.909
Arrival systolic BP	140.29 ± 32.61	137.97 ± 31.30	0.627
Arrival diastolic BP	75.97 ± 18.11	76.79 ± 18.13	0.761

RBC, red blood cell; WBC, white blood cell; ESR, erythrocyte sedimentation rate; AST, aspartate aminotransferase; ALT, alanine aminotransferase; ALP, alkaline phosphatase; BUN, blood urea nitrogen; HbA1c, hemoglobin A1c; HDL, high-density lipoprotein; LDL, low-density lipoprotein; TSH, thyroid-stimulating hormone; FDP, fibrinogen degradation production; BP, blood pressure; ETC* includes malignant neoplasm (4), brain abscess (3), tetanus (3), acute transverse myelitis (2), IgG4-related disease (1), injury of cervical spinal cord (1), halogenated insecticides (1), anoxic brain damage (1), Guillain-Barre syndrome (1), Creutzfeldt-Jakob disease (1), myocardial infarction (1).

This study compared patients who died after hospitalization in the neurology department based on their main diagnosis (non-ischemic stroke vs. ischemic stroke) (Table 2). The mean age was 69.45 ± 15.84 years for non-ischemic stroke patients and 76.44 ± 10.36 years for ischemic stroke patients (*p* < 0.001). Sepsis (26.8 vs. 13.7%), pneumonia (12.5 vs. 6.1%), acute kidney injury (12.5 vs. 1.5%), and status epilepticus (12.5 vs. 0%) were direct causes of death significantly more frequently in the non-ischemic stroke group than in the ischemic stroke group (*p* < 0.001). Consciousness status at the time of hospital visit was not significantly different between the two groups. However, semicoma (18.2 vs. 7.9%) and coma (7.3 vs. 3.2%) status were significantly more common in the non-ischemic stroke group than in the ischemic stroke group (*p* = 0.056). Atrial fibrillation (13.0 vs. 34.6%; *p* = 0.003) and a history of stroke (11.1 vs. 29.1%; *p* = 0.009) were significantly more common in the ischemic stroke group than in the non-ischemic stroke group. There were no significant differences in the rates of tracheal intubation or ICU admission between the two groups. The non-ischemic stroke group had significantly higher C-reactive protein (76.76 ± 88.32 vs. 29.74 ± 51.49; *p* < 0.001) and significantly lower albumin (3.56 ± 0.72 vs. 3.80 ± 0.73; *p* = 0.035) levels than did the ischemic stroke group.

**Table 2:** Comparison of patients with ischemic stroke and those without ischemic stroke.

	Without ischemic stroke (*n* = 56)	With ischemic stroke (*n* = 131)	*p* value
Age, mean ± standard deviation (years)	69.45 ± 15.84	76.44 ± 10.36	<0.001
Male	32 (57.1)	64 (48.9)	0.299
Direct cause of death			
Ischemic stroke	1 (1.8)	67 (51.1)	<0.001
Sepsis	15 (26.8)	18 (13.7)	
Pneumonia	7 (12.5)	8 (6.1)	
Cerebral hemorrhage	2 (3.6)	17 (13.0)	
Acute kidney injury	7 (12.5)	2 (1.5)	
Status epilepticus	7 (12.5)	0	
ETC	17 (30.3)	19 (14.5)	
Length of hospital day	17.25 ± 21.52	11.31 ± 22.87	0.101
Mental status on admission			
Alert	18 (32.7)	38 (30.2)	0.056
Drowsy	7 (12.7)	36 (28.6)	
Stupor	16 (29.1)	38 (30.2)	
Semicoma	10 (18.2)	10 (7.9)	
Coma	4 (7.3)	4 (3.2)	
Hypertension	33 (58.9)	90 (69.8)	0.151
Diabetes mellitus	18 (32.1)	43 (33.6)	0.847
Atrial fibrillation	7 (13.0)	44 (34.6)	0.003
Smoking	14 (25.0)	36 (27.5)	0.726
Dyslipidemia	7 (12.7)	8 (6.4)	0.157
Alcohol	18 (32.1)	28 (21.4)	0.117
Body mass index (kg/m^2^)	22.60 ± 3.99	22.83 ± 3.33	0.898
Previous stroke history	6 (11.1)	37 (29.1)	0.009
Pre-existing disease			
Cancer	8 (14.3)	22 (16.8)	0.669
Circulatory	8 (14.3)	27 (20.6)	0.310
Nervous	7 (12.5)	15 (11.5)	0.838
Dementia	4 (7.1)	14 (10.7)	0.452
Genitourinary	13 (23.2)	18 (13.7)	0.111
Musculoskeletal, Connective tissue	8 (14.3)	17 (13.0)	0.810
Respiratory	9 (16.1)	11 (8.4)	0.120
Digestive	6 (10.7)	10 (7.6)	0.490
Endocrine	4 (7.1)	3 (2.3)	0.109
Others	17 (30.4)	23 (17.6)	0.051
Tracheal intubation	43 (76.8)	103 (80.5)	0.570
Intensive care unit care	44 (84.6)	108 (86.4)	0.756
White matter hyperintensity (Fazekas scale)			
0	1 (2.4)	1 (1.2)	0.118
1	19 (45.2)	20 (24.7)	
2	9 (21.4)	24 (29.6)	
3	13 (31.0)	36 (44.4)	
Microbleeds			
None	2 (4.9)	2 (2.1)	0.091
Mild	8 (19.5)	6 (6.3)	
Moderate	17 (41.5)	46 (48.4)	
Severe	14 (34.1)	41 (43.2)	
Laboratory finding			
WBC ($10^{3}$/μL)	12.61 ± 6.56	10.74 ± 4.38	0.054
Hemoglobin (g/dL)	11.94 ± 2.10	12.09 ± 2.66	0.712
Platelet ($10^{3}$/μL)	209.09 ± 104.55	218.31 ± 94.36	0.555
ESR (mm/h)	37.38 ± 30.12	29.17 ± 25.08	0.077
Sodium (mEq/L)	136.00 ± 5.63	137.72 ± 4.82	0.036
Potassium (mEq/L)	4.22 ± 0.89	4.41 ± 2.55	0.576
C-reactive protein (mg/dL)	76.76 ± 88.32	29.74 ± 51.49	<0.001
Albumin (g/dL)	3.56 ± 0.72	3.80 ± 0.73	0.035
AST (U/L)	45.64 ± 27.03	48.17 ± 79.88	0.818
ALT (U/L)	32.29 ± 25.00	33.12 ± 46.68	0.900
ALP (U/L)	134.38 ± 154.39	100.04 ± 66.91	0.236
BUN (mg/dL)	29.85 ± 20.13	22.96 ± 13.34	0.022
Creatinine (mg/dL)	3.30 ± 15.44	1.10 ± 1.08	0.301
HbA1c (%)	6.31 ± 1.31	6.26 ± 1.24	0.845
Triglycerides (mg/dL)	106.67 ± 43.54	134.68 ± 106.11	0.370
HDL (mg/dL)	33.08 ± 15.85	38.39 ± 14.69	0.249
LDL (mg/dL)	79.67 ± 38.20	92.69 ± 41.58	0.308
Total cholesterol (mg/dL)	128.08 ± 50.27	150.69 ± 47.94	0.118
Calcium (mg/dL)	8.25 ± 1.89	8.55 ± 0.96	0.365
TSH (μIU/mL)	1.88 ± 2.19	3.25 ± 7.83	0.297
Free T4 (mg/dL)	15.62 ± 4.50	17.01 ± 7.53	0.297
Fibrinogen (mg/dL)	346.78 ± 141.90	333.43 ± 94.71	0.634
FDP (ug/mL)	19.29 ± 29.99	26.81 ± 84.13	0.623
D-dimer (mg/L FEU)	3.77 ± 4.36	3.90 ± 6.05	0.892
Arrival systolic BP	130.91 ± 30.90	142.74 ± 31.77	0.021
Arrival diastolic BP	71.75 ± 15.13	78.42 ± 18.93	0.022

RBC, red blood cell; WBC, white blood cell; ESR, erythrocyte sedimentation rate; AST, aspartate aminotransferase; ALT, alanine aminotransferase; ALP, alkaline phosphatase; BUN, blood urea nitrogen; HbA1c, hemoglobin A1c; HDL, high-density lipoprotein; LDL, low-density lipoprotein; TSH, thyroid-stimulating hormone; FDP, fibrinogen degradation production; BP, blood pressure.

This study identified the factors associated with those who died from ischemic stroke among those who died (Table 3). Univariate logistic regression analysis showed significant differences in age (OR 1.044, 95% CI 1.017–1.072, *p* = 0.001), previous stroke (OR 3.289, 95% CI 1.296–8.344, *p* = 0.012), CRP (OR 0.990, 95% CI 0.986–0.995, *p* < 0.001), albumin (OR 1.559, 95% CI 1.017–2.390, *p* = 0.042). Multivariate logistic regression analysis identified that age (aOR 1.041, 95% CI 1.013–1.071, *p* = 0.004), previous stroke (aOR 3.839, 95% CI 1.400–10.528, *p* = 0.009) were significant factors associated with the deaths of ischemic stroke, but CRP (aOR 0.993, 95% CI 0.986–0.999, *p* = 0.02) was associated with the deaths of other causes.

**Table 3:** Univariate and multivariate analyses of factors associated with death of ischemic stroke.

Variables	Univariate analysis		Multivariate analysis	
	Crude OR (95% CI)	*p* value	Adjusted OR (95% CI)	*p* value
Age	1.044 (1.017–1.072)	0.001	1.041 (1.013–1.071)	0.004
Previous stroke	3.289 (1.296–8.344)	0.012	3.839 (1.400–10.528)	0.009
CRP	0.990 (0.986–0.995)	<0.001	0.993 (0.986–0.999)	0.02
Albumin	1.559 (1.017–2.390)	0.042	1.910 (0.936–3.898)	0.076

OR and *p*-value by logistic regression using Firth’s penalized maximum likelihood method.

CI, confidence intervals; CRP, C-reactive protein.

Moreover, this study conducted univariate and multivariate logistic regression analysis to identify the factors associated with the deaths of sepsis and pneumonia (Supplementary Tables 2, 3). In sepsis, Univariate logistic regression analysis showed significant differences in musculoskeletal or connective tissue diseases (OR 3.512, 95% CI 1.372–8.988, *p* = 0.009), CRP (OR 1.008, 95% CI 1.003–1.013, *p* = 0.002), albumin (OR 0.546, 95% CI 0.315–0.944, *p* = 0.03), tracheal intubation (OR 5.108, 95% CI 1.151–22.670, *p* = 0.032). Multivariate logistic regression analysis identified that musculoskeletal or connective tissue diseases (OR 3.006, 95% CI 1.075–8.409, *p* = 0.036), tracheal intubation (OR 4.558, 95% CI 1.001–20.761, *p* = 0.05) were significant factors associated with the deaths of sepsis. In pneumonia, Univariate logistic regression analysis showed significant differences in previous stroke history (OR 3.467, 95% CI 1.127–10.663, *p* = 0.03), Intensive care unit care (OR 0.248, 95% CI 0.067–0.918, *p* = 0.037). Multivariate logistic regression analysis identified that previous stroke history (OR 4.763, 95% CI 1.310–17.316, *p* = 0.018) was a significant factor associated with the deaths of pneumonia.

Furthermore, the patients who died after neurological hospitalization were compared between the cancer and non-cancer groups (Table 4). Musculoskeletal and connective tissue diseases (15.9% vs. 0%; *p* = 0.016) were significantly more common in the non-cancer group. In addition, the non-cancer group had significantly higher hemoglobin (12.20 ± 2.51 vs. 11.21 ± 2.29; *p* = 0.046), platelet (221.93 ± 93.76 vs. 182.90 ± 110.24; *p* = 0.045), and high-density lipoprotein (39.19 ± 15.26 vs. 30.38 ± 10.06; *p* = 0.029) than did the cancer group.

**Table 4:** Comparison of patients with cancer and without cancer.

	Without cancer (*n* = 157)	With cancer (*n* = 30)	*p* value
Age, mean ± standard deviation (years)	74.50 ± 12.96	73.57 ± 10.91	0.713
Male	78 (49.7)	18 (60)	0.300
With ischemic stroke	109 (69.4)	22 (73.3)	0.669
Principal diagnosis classification			
Ischemic stroke	109 (69.4)	22 (73.3)	0.762
Seizure	17 (10.8)	2 (6.7)	
Encephalitis and encephalopathy	16 (10.2)	2 (6.7)	
ETC*	15 (9.6)	4 (13.3)	
Direct cause of death			
Ischemic stroke	54 (43.5)	14 (51.9)	0.340
Sepsis	28 (22.6)	5 (18.5)	
Pneumonia	10 (8.1)	5 (18.5)	
Cerebral hemorrhage	17 (13.7)	2 (7.4)	
Acute kidney injury	9 (7.3)	0	
Status epilepticus	6 (4.8)	1 (3.7)	
Length of hospital day	13.32 ± 22.83	12.10 ± 21.48	0.790
Hypertension	105 (67.7)	18 (60.0)	0.411
Diabetes mellitus	54 (34.8)	7 (24.1)	0.261
Atrial fibrillation	45 (29.8)	6 (20.0)	0.276
Smoking	44 (28.0)	6 (20.0)	0.363
Dyslipidemia	14 (9.3)	1 (3.3)	0.278
Alcohol	38 (24.2)	8 (26.7)	0.774
Body mass index (kg/ss)	22.83 ± 3.32	22.43 ± 4.62	0.854
Previous stroke history	34 (22.5)	9 (30.0)	0.379
Pre-existing disease			
Circulatory	31 (19.7)	4 (13.3)	0.409
Nervous	18 (11.5)	4 (13.3)	0.759
Dementia	17 (10.8)	1 (3.3)	0.202
Genitourinary	29 (18.5)	2 (6.7)	0.111
Musculoskeletal, Connective tissue	25 (15.9)	0	0.016
Respiratory	19 (12.1)	1 (3.3)	0.154
Digestive	16 (10.2)	0	0.067
Endocrine	6 (3.8)	1 (3.3)	0.897
Others	34 (21.7)	6 (20.0)	0.839
Tracheal intubation	125 (81.2)	21 (70.0)	0.167
Intensive care unit care	129 (86.0)	23 (85.2)	0.911
White matter hyperintensity (Fazekas scale)			
0	2 (1.9)	0	0.678
1	31 (29.8)	8 (42.1)	
2	28 (26.9)	5 (26.3)	
3	43 (41.3)	6 (31.6)	
Microbleeds			
None	3 (2.6)	1 (5.0)	0.437
Mild	11 (9.5)	3 (15.0)	
Moderate	57 (49.1)	6 (30.0)	
Severe	45 (38.8)	10 (50.0)	
Laboratory finding			
WBC ($10^{3}$/μl)	11.49 ± 5.24	10.37 ± 4.92	0.283
Hemoglobin (g/dL)	12.20 ± 2.51	11.21 ± 2.29	0.046
Platelet ($10^{3}$/μl)	221.83 ± 93.76	182.90 ± 110.24	0.045
ESR (mm/hr)	32.35 ± 27.52	27.83 ± 23.26	0.400
Sodium (mEq/L)	137.27 ± 5.09	136.83 ± 5.41	0.670
Potassium (mEq/L)	4.22 ± 0.85	5.06 ± 5.12	0.380
C-reactive protein (mg/dL)	44.87 ± 71.3	39.34 ± 49.49	0.685
Albumin (g/dL)	3.71 ± 0.77	3.83 ± 0.51	0.398
AST (U/L)	48.54 ± 73.42	41.57 ± 29.82	0.610
ALT (U/L)	33.60 ± 43.86	29.07 ± 23.86	0.583
ALP (U/L)	116.80 ± 11.56	80.75 ± 30.53	0.204
BUN (mg/dL)	25.54 ± 16.70	22.37 ± 11.11	0.320
Creatinine (mg/dL)	1.92 ± 9.26	0.86 ± 0.29	0.529
HbA1c (%)	6.31 ± 1.27	6.08 ± 1.22	0.438
Triglycerides (mg/dL)	127.53 ± 85.80	149.88 ± 159.03	0.421
HDL (mg/dL)	39.19 ± 15.26	30.38 ± 10.06	0.029
LDL (mg/dL)	90.10 ± 39.30	96.19 ± 51.11	0.657
Total cholesterol (mg/dL)	148.52 ± 48.20	143.56 ± 52.05	0.711
Calcium (mg/dL)	8.36 ± 1.47	8.81 ± 0.62	0.260
TSH (μIU/mL)	3.20 ± 7.36	1.27 ± 0.79	0.213
Free T4 (mg/dL)	16.64 ± 7.26	16.42 ± 4.21	0.889
Fibrinogen (mg/dL)	342.83 ± 109.53	307.91 ± 114.22	0.221
FDP (μg/mL)	17.14 ± 27.45	57.24 ± 157.02	0.257
D-dimer (mg/L FEU)	3.56 ± 4.61	5.49 ± 9.14	0.284

RBC, red blood cell; WBC, white blood cell; ESR, erythrocyte sedimentation rate; AST, aspartate aminotransferase; ALT, alanine aminotransferase; ALP, alkaline phosphatase; BUN, blood urea nitrogen; HbA1c, hemoglobin A1c; HDL, high-density lipoprotein; LDL, low-density lipoprotein; TSH, thyroid-stimulating hormone; FDP, fibrinogen degradation production; BP, blood pressure; ETC* comprises malignant neoplasm (4), brain abscess (3), tetanus (3), acute transverse myelitis (2), IgG4-related disease (1), injury of cervical spinal cord (1), halogenated insecticides (1), anoxic brain damage (1), Guillain-Barre syndrome (1), Creutzfeldt-Jakob disease (1), myocardial infarction (1).

## 4 Discussion

This retrospective study evaluated the characteristics of patients who died as inpatients in a neurology department. With the aim of identifying ways to reduce the risk of death in patients admitted to a neurology department, the present study identified the neurological diseases treated at a regional tertiary hospital in South Korea, analyzed the patients’ principal diagnoses and direct causes of death, and determined the causes of death.

The number of inpatients was similar each year; however, the number of deaths was particularly high in 2019. Of the 187 deaths that occurred between 2013 and 2019, 18 (9.6%) patients were diagnosed with dementia. Of the 33 deaths in 2019, four (12.1%) patients had a diagnosis of dementia, which was higher than the mean death rate (9.6%). Statistics Korea reported that Alzheimer’s disease-related deaths increased in 2019 compared to the deaths in the previous year; the results of the present study agree with this report (11). We suspected that this was due to an increase in the mean age and diagnostic rate of dementia compared to those in the past.

Moreover, the present study revealed the highest number and proportion of ischemic stroke-related deaths. Diaz et al. (4) examined neurology inpatients in Uganda and found that stroke was the most common cause of death. In the present study, the ICU hospitalization rate was 86.4% (108/125) in patients whose primary diagnosis was ischemic stroke, whereas the ICU hospitalization rates were 88.2% (15/17), 83.3% (15/18), and 82.4% (14/17) in patients whose primary diagnosis was seizures, meningoencephalitis, and encephalopathy, respectively. The mean hospitalization duration was longer in patients with seizures than in those with ischemic stroke. Based on these results, we speculated that ischemic strokes are more severe than other neurological conditions that occlude large vessels or involve the brainstem, leading to death.

Moreover, sex-based classification revealed several noteworthy findings. The principal diagnosis was not significantly different between men and women; however, the direct cause of death differed significantly (*p* = 0.001). In particular, when the principal diagnosis was ischemic stroke, it was a more common direct cause of death in women than in men (39.1% vs. 62.7%), whereas cerebral hemorrhage (23.4% vs. 3.0%) and pneumonia (9.4% vs. 3.0%) were more common causes of death in men than in women. These results suggest that sex and other factors may affect the clinical course of ischemic stroke. Firstly, for ischemic stroke, the incidence of atrial fibrillation was higher in women than in men. Kapral et al. (12) compared the clinical characteristics of stroke onset between sexes and reported that the prevalence of atrial fibrillation was higher in women than in men; they inferred that it could increase the risk of cardioembolic stroke and frequency of cardioembolic stroke in women than in men (12). In the present study, the prevalence of atrial fibrillation and cardioembolic stroke was also higher in women than in men (12.5% vs. 19.4%). Cardioembolic stroke is clinically more severe than other ischemic stroke mechanisms (13); therefore, it can affect the fatality rate of ischemic stroke. As a result, the incidence of ischemic stroke as a direct cause of death is believed to be higher in women than in men. In addition, female sex could increase the risk of ischemic stroke due to biological differences between the sexes (sex hormones), sex-related factors (lack of stroke awareness and social support), and a higher frequency of atypical stroke symptoms (mental change, general weakness, fatigue, and headache) (14). Moreover, the incidence of asymptomatic symptoms was higher in female patients (67.2%) than in male patients (61.0%) diagnosed with ischemic stroke in present study, which can lead to a high mortality rate. Secondly, for cerebral hemorrhage, Ögren et al. (15) reported a decreased risk of intracranial hemorrhage after ischemic stroke in an old female patient. Therefore, because the mean age of women was higher than that of men in the present study, it can be assumed that the frequency of ischemic hemorrhage was lower. Lastly, more men died due to status epilepticus than did women in this report (5.2% vs. 2.2%), and Choi et al. (16) reported that the male sex was a poor prognostic factor for status epilepticus.

The direct cause of death differed between patients with non-ischemic stroke and those with ischemic stroke. Non-ischemic stroke patients showed a higher incidence of sepsis (15 patients, 26.8%), followed by pneumonia (seven patients, 12.5%), acute kidney injury (seven patients, 12.5%), status epilepticus (seven patients, 12.5%), and cerebral hemorrhage (two patients, 3.6%). The direct causes of death in patients with ischemic stroke were ischemic stroke (67 patients, 51.1%), sepsis (18 patients, 13.7%), cerebral hemorrhage (17 patients, 13.0%), and pneumonia (eight patients, 6.1%). Considering that C-reactive protein levels were significantly higher in the non-ischemic stroke group, infectious diseases such as sepsis and pneumonia could be directly responsible for a higher proportion of deaths. Furthermore, we observed significant differences in stroke history. Stroke history can be a risk factor for stroke recurrence, as reported frequently in previous studies; moreover, this study showed that stroke history was significantly higher in patients with ischemic stroke than in those with non-ischemic stroke (17).

Previous studies have suggested the cardiovascular or neurological factors associated with death after stroke (18). The results of the present study revealed that the proportion of patients with atrial fibrillation was higher in the ischemic stroke group than in the non-ischemic stroke group. Blood pressure at the time of admission was also higher in the ischemic stroke group. However, the rate of hypertension diagnosis was not significantly different between the two groups. Blood pressure may have been affected by numerous variables because it was measured only once at the time of admission. Therefore, it was difficult to determine whether these patients had hypertension. However, appropriate treatment of hypertension has been reported to significantly reduce the incidence of stroke in older adults (19). In the present study, patients admitted to the neurology department for ischemic stroke had a higher blood pressure at the time of admission. Therefore, we need to perform close blood pressure management by repeatedly measuring blood pressure to achieve an expected decrease in the incidence of stroke. Wafa et al. (20) had also analyzed that the rate of stroke increased because of population aging and improved survival rates. In our study, old age was also identified as a factor highly associated with death from ischemic stroke. Therefore, it can be judged that there is a high possibility that elderly stroke patients will progress to a more serious course, and careful management is needed both before and after the stroke occurs.

Sepsis and pneumonia are the most common direct causes of death following ischemic stroke, which implies that the infection is important for the course after stroke. However, diagnosis of infection after stroke is difficult due to the fact that fever and inflammation reaction, which are major signs of infection, can be also caused by stroke, and it is difficult to identify symptoms due to drugs or worsening neurological condition (21). Therefore, this study analyzed patients who died from sepsis and pneumonia and found the associated factors. First, in the case of sepsis, having musculoskeletal or connective tissue diseases as an underlying disease and performing tracheal intubation were significant factors. Also, in the case of pneumonia, previous stroke history was a meaningful factor. As a result, careful attention is needed to ensure that post-stroke infection does not lead to death in patients with such factors analyzed in this study, as well as factors previously known to be associated with infection after stroke; National Institutes of Health Stroke Scale score, prestroke dependence, atrial fibrillation, congestive heart failure, diabetes, low Glasgow Coma Scale on admission, and chronic obstructive pulmonary disease (22).

This study had several limitations. First, this study evaluated patients from a single tertiary hospital, which might have led to a selection bias owing to the predominance of patients with severe diseases. As the study participants were deceased patients, most might have sought medical attention at the hospital because of the severity of their conditions. Second, because this research targeted only the deceased, future research will require comparative studies between survivors and deceased after hospitalization. This research might help to analyze contributors to mortality rates. Moreover, because the main diagnoses were classified according to the ICD criteria, we could not review the details. Nevertheless, we could check the proportions of the main diagnoses and direct causes of death among neurology inpatients in a single institution and understand the characteristics of all deceased patients. The results of this study revealed that ischemic stroke was the main diagnosis and direct cause of death in most patients.

Thus, it is necessary to actively treat inpatients who are likely to experience an ischemic stroke at an early stage; treatment should always be performed with consideration of the risk of a cerebral hemorrhage after an ischemic stroke. Regarding the direct causes of death, the proportion of patients with sepsis and pneumonia was high after ischemic stroke. Therefore, it is necessary to manage environments that can cause infections after hospitalization. If there are signs and symptoms of infection, active treatment and management are necessary to evaluate the possibility of sepsis using quick SOFA (qSOFA) or SOFA scoring. In addition, our study is meaningful because its findings provide evidence for the management of other diseases.

## Data Availability

The raw data supporting the conclusions of this article will be made available by the authors, without undue reservation.
