# Supplementary material for: Characteristics and direct causes of death in severely ill patients admitted to a neurology department
Source: Front Neurol. 2024 Dec 13;15:1386403. doi: 10.3389/fneur.2024.1386403 (PMC11681426; doi:10.3389/fneur.2024.1386403)
Supplement: Supplementary file 1 [file Table_1.DOCX]

***Supplementary Material***

# Supplementary Figures and Tables

**Supplementary Table 1.** Baseline characteristics of the patients

| Variables | *(n=187)* |
| --- | --- |
| Age, mean ± standard deviation (years) | 74.35 ± 12.63 |
| Man | 96 (51.3) |
| Principal diagnosis classification |  |
| Ischemic stroke | 131 (70.1) |
| Seizure | 19 (10.2) |
| Encephalitis and encephalopathy | 18 (9.5) |
| ETC* | 19 (10.2) |
| Direct cause of death |  |
| Ischemic stroke | 68 (36.4) |
| Sepsis | 33 (17.6) |
| Pneumonia | 15 (8.0) |
| Cerebral hemorrhage | 19 (10.2) |
| Acute kidney injury | 9 (4.8) |
| Status epilepticus | 7 (3.7) |
| ETC | 36 (19.3) |
| Length of hospital day | 13.13 ± 22.57 |
| Hypertension | 123 (65.8) |
| Diabetes mellitus | 61 (32.6) |
| Atrial fibrillation | 51 (27.3) |
| Smoking | 50 (26.7) |
| Dyslipidemia | 15 (8.0) |
| Alcohol | 46 (24.6) |
| Body mass index (kg/m^2^) | 22.78 ± 3.39 |
| Previous stroke history | 43 (23.0) |
| Pre-existing disease |  |
| Cancer | 30 (16.1) |
| Circulatory | 35 (18.7) |
| Nervous | 22 (11.8) |
| Dementia | 18 (9.6) |
| Genitourinary | 31 (16.6) |
| Musculoskeletal, Connective tissue | 25 (13.4) |
| Respiratory | 20 (10.7) |
| Digestive | 16 (8.6) |
| Endocrine | 7 (3.7) |
| Others | 40 (21.4) |
| Tracheal intubation | 146 (78.1) |
| Intensive care unit care | 152 (81.3) |
| White matter hyperintensity (Fazekas scale) |  |
| 0 | 2 (1.1) |
| 1 | 39 (20.9) |
| 2 | 33 (17.6) |
| 3 | 49 (26.2) |
| Microbleeds |  |
| None | 4 (2.1) |
| Mild | 14 (7.5) |
| Moderate | 63 (33.7) |
| Severe | 55 (29.4) |

ETC* comprises malignant neoplasm (4), brain abscess (3), tetanus (3), acute transverse myelitis (2), IgG4-related disease (1), injury of cervical spinal cord (1), halogenated insecticides (1), anoxic brain damage (1), Guillain-Barre syndrome (1), Creutzfeldt-Jakob disease (1), myocardial infarction (1)

# Supplementary Figures and Tables

**Supplementary Table 2.** Univariate and multivariate analyses of factors associated with death of sepsis

| Variables | Univariate analysis |  | Multivariate analysis |  |
| --- | --- | --- | --- | --- |
|  | Crude OR (95% CI) | *P value* | Adjusted OR (95% CI) | *P value* |
| Musculoskeletal or Connective tissue | 3.512 (1.372 - 8.988) | 0.009 | 3.006 (1.075 - 8.409) | 0.036 |
| CRP | 1.008 (1.003 - 1.013) | 0.002 | 1.005 (0.998 - 1.011) | 0.137 |
| Albumin | 0.546 (0.315 - 0.944) | 0.03 | 0.621 (0.280 - 1.374) | 0.24 |
| Tracheal intubation | 5.108 (1.151 - 22.670) | 0.032 | 4.558 (1.001 - 20.761) | 0.05 |

OR and P-value by logistic regression using Firth’s penalized maximum likelihood method

CI, confidence intervals; CRP, C-reactive protein

# Supplementary Figures and Tables

**Supplementary Table 3.** Univariate and multivariate analyses of factors associated with death of pneumonia

| Variables | Univariate analysis |  | Multivariate analysis |  |
| --- | --- | --- | --- | --- |
|  | Crude OR (95% CI) | *P value* | Adjusted OR (95% CI) | *P value* |
| Previous stroke history | 3.467 (1.127 - 10.663) | 0.03 | 4.763 (1.310 - 17.316) | 0.018 |
| Tracheal intubation | 0.341 (0.111 - 1.045) | 0.06 | 0.310 (0.086 - 1.121) | 0.074 |
| Intensive care unit care | 0.248 (0.067 - 0.918) | 0.037 | 0.274 (0.065 - 1.149) | 0.077 |

OR and P-value by logistic regression using Firth’s penalized maximum likelihood method

CI, confidence intervals
